# Glanuloplasty with Oral Mucosa Graft following Total Glans Penis Amputation

**DOI:** 10.1155/2014/671303

**Published:** 2014-08-12

**Authors:** Kwaku Appiah, George Amoah, Roland Azorliade, Kofi Gyasi-Sarpong, Ken Aboah, Douglas Arthur, Baah Nyamekye, Kwaku Otu-Boateng, Patrick Maison, Benjamin Twumasi-Frimpong, Issac Opoku Antwi, Edwin Yenli

**Affiliations:** ^1^Department of Surgery, Komfo Anokye Teaching Hospital, Kumasi, Ghana; ^2^Department of Surgery, School of Medical Sciences, College of Health Sciences, KNUST, Kumasi, Ghana

## Abstract

This is a report on the technique of neoglans reconstruction in a patient with amputated glans penis following guillotine neonatal circumcision. A 4 cm long and 2 cm wide lower lip oral mucosa graft was harvested and used to graft the distal 2 cm of the corporal bodies after 2 cm of the distal penile skin had been excised. One edge of the lower lip oral mucosa graft was anastomosed to the urethral margins distally and proximally to the skin. At six months of followup, patient had both satisfactory cosmetic and functional outcomes.

## 1. Introduction

Circumcision is the commonest operation performed on young boys [1]. Like any other operation, it is not without complications and that can range from trivial to the most tragic [2]. In Ghana, circumcision is regarded as a customary ritual and is mostly done by nurses, the traditional circumcisionist (Wanzam) with medical doctors being involved less often. This is probably due to the high patient to doctor ratio making the doctors shirk this responsibility to untrained personnel. As a result of this, many complications arise ranging from minor injuries to catastrophic penile amputations which are usually not reported immediately. In our center, unpublished data on 72 cases of circumcision injuries over a two-year period reveals that urethrocutaneous fistula accounts for 77.8% with penile amputation accounting for 6.9% of all injuries. Of the 72 circumcision injuries, nurses were responsible for 77.8% with the traditional circumcisionist involved in 20.8% and medical doctors being involved in 8.3% of times. In a similar study in Nigeria by Okeke et al., complications of circumcision tended to be more likely with nurses than with doctors or traditional circumcisionists, though this observation did not reach statistical significance. In this study, 320 out of 370 male children had circumcision and complications occurred in 65 (20.2%) including redundant foreskin in 35 (53.8%), excessive loss of foreskin in 16 (24.6%), 11 (16.9%) had skin bridges, and 2 (3.1%) sustained amputation of the glans penis while 1 (1.5%) had a buried penis.

Penile amputation at any level is rare but it is the most seriously reported complication of circumcision [3].

The management of penile glans amputation depends on the duration before presentation and the acute phase management usually involves autotransplantation [3–10].

We present a case report of the use of oral mucosa graft in neoglans reconstruction in a boy with total glans penis amputation following circumcision which can guarantee long term urethral opening and acceptable cosmesis and prevent further shortening of the penis.

Traditionally oral mucosa grafts have been used in urethral reconstruction. In recent times, it has been used for resurfacing of the glans penis or reconstructing a neoglans penis after partial or total glansectomy for penile cancers [11].

From online literature search and to the best of our knowledge, it appears that this procedure has not been utilized in the management of traumatic glans penis amputations following circumcision in late presentations as has been done in cancer associated glansectomies.

## 2. Case Report

This is a case of a 3-year-old boy that had total glans penis amputation at the time of guillotine circumcision by traditional circumcisionist (locally referred to as Wanzam) in the neonatal period. The circumcision injury had completely healed at the time of presentation. The mother brought him to our facility three years after the injury on account of difficulty in urination and a disfigured penis. The boy passed a poor stream of urine with straining.

**Figure 1:**
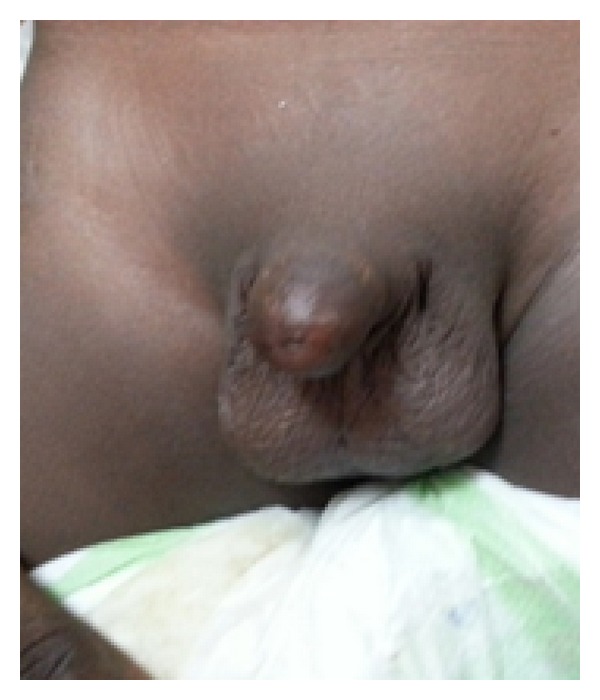
Total glans penis amputation with meatal stenosis from scarring.

On physical examination, he was a well-looking boy with normal growth for his age. The glans penis and the coronal sulcus were absent. He had a pinhole urethral meatus with scaring at the distal end (Figure 1) but adequate penile stump length.

His postvoid residual urine volume was 80 mls. The bladder wall was thickened but there was no hydronephrosis on ultrasound scan. His renal function was normal and urine culture did not isolate any organism.

From the clinical presentation above, the goals of management were to achieve a cosmetically acceptable neoglans penis, achieve a long term widely patent urethral opening, and prevent further penile shortening.

## 3. Description of the Surgical Procedure

**Figure 2:**
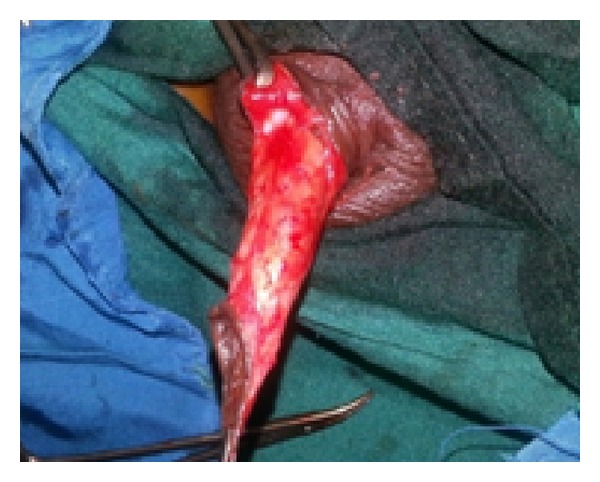
Penis degloved leaving scar tissue at the end which was excised.

*Step  1*.  Surgery involved a circumcising incision, degloving of the penile skin, and excision of the scar tissue at the distal end exposing the corporeal bodies in the process (Figure 2).

**Figure 3:**
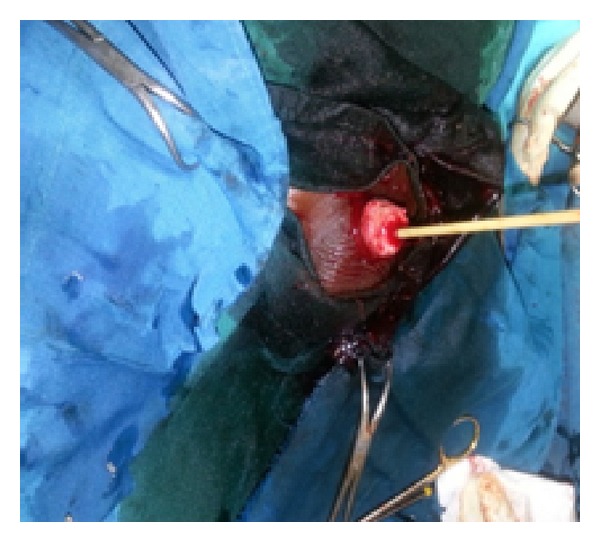
Penile skin shortened by 2 cm and sutured into place.

*Step  2*. The urethral end was identified and catheterized with a size 6 fr urethral catheter. The distal penile skin was shortened by 2 cm leaving raw surfaces of the corporeal bodies distally and sutured into place with vicryl 3/0 (Figure 3).

**Figure 4:**
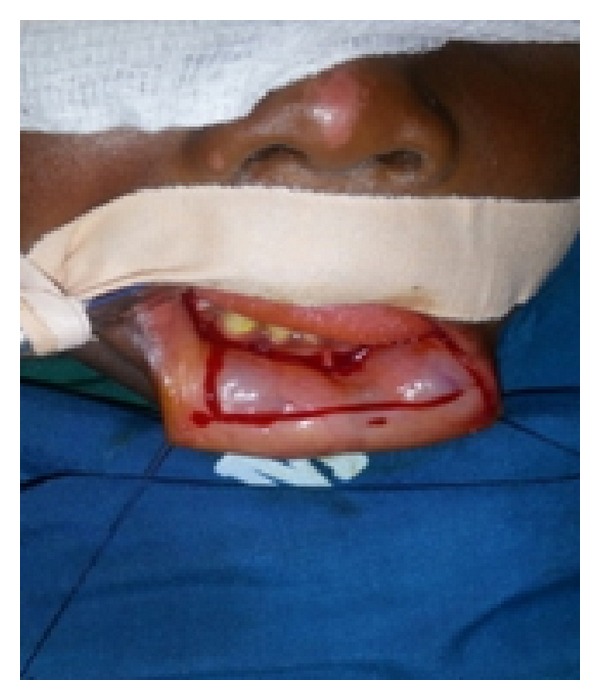
Oral mucosa graft being harvested from the lower lip.

**Figure 5:**
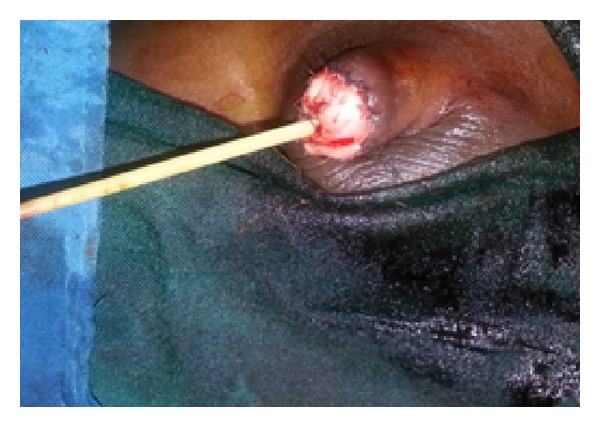
Oral mucosa used to graft the raw area of the corporeal bodies.

*Step  3*. A 4 cm long, 2 cm wide lower lip oral mucosa graft (Figure 4) was harvested and tiny fenestrations were made into it. One edge of the graft was sutured to the penile skin proximally and the other edge was anastomosed to the urethral mucosal margins (Figure 5). Anchoring sutures were applied to stabilize the graft on the corporeal bodies.

Postoperatively, closed wound dressing with Vaseline gauze was applied to the recipient site and only changed on the fifth day. It was then changed at four-day intervals on two occasions.

The urethral catheter was taken out on the 14th day after surgery.

## 4. Results

**Figure 6:**
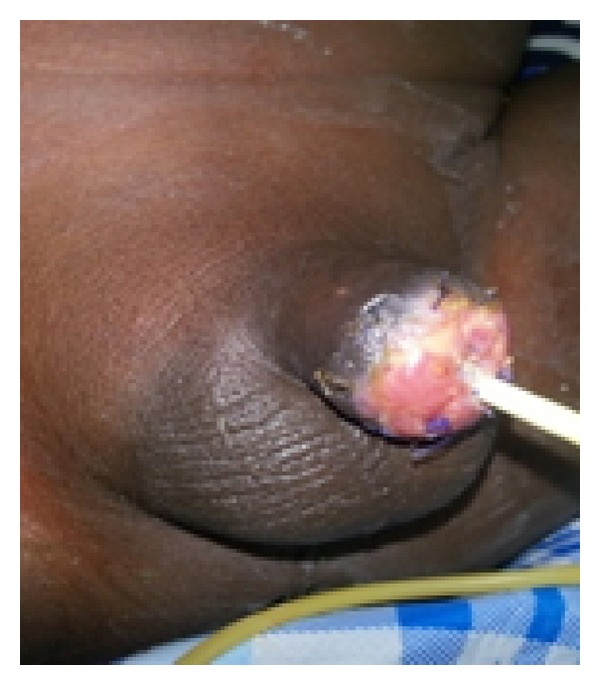
Glans at two weeks following surgery.

**Figure 7:**
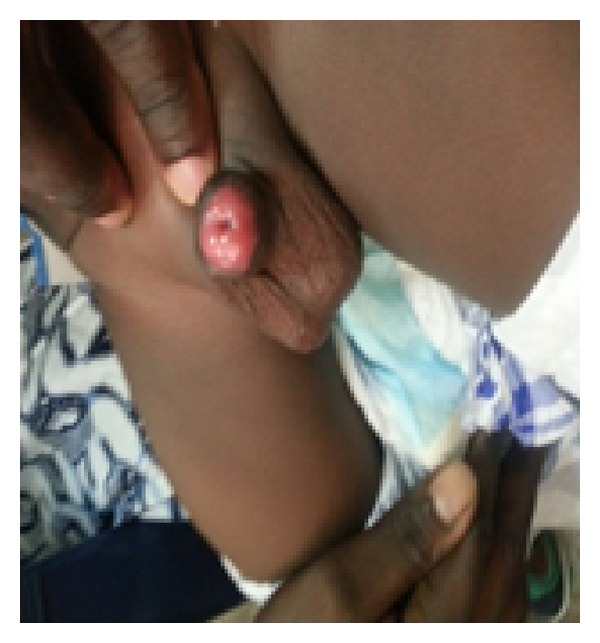
Glans at eight weeks following surgery.

**Figure 8:**
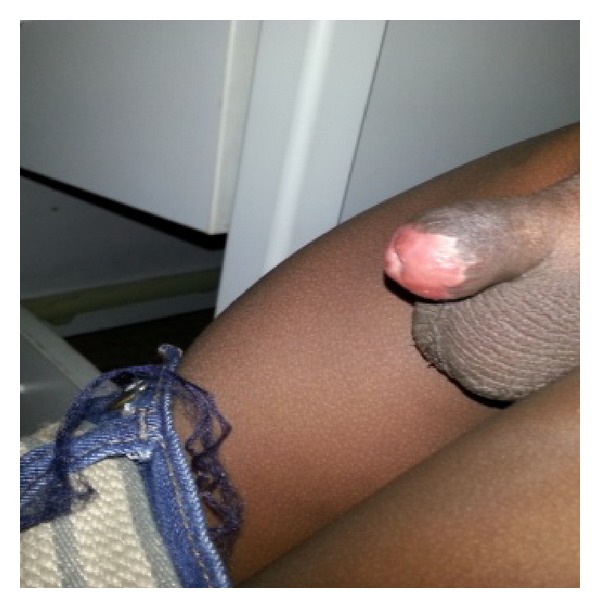
Glans penis at six months (lateral view).

**Figure 9:**
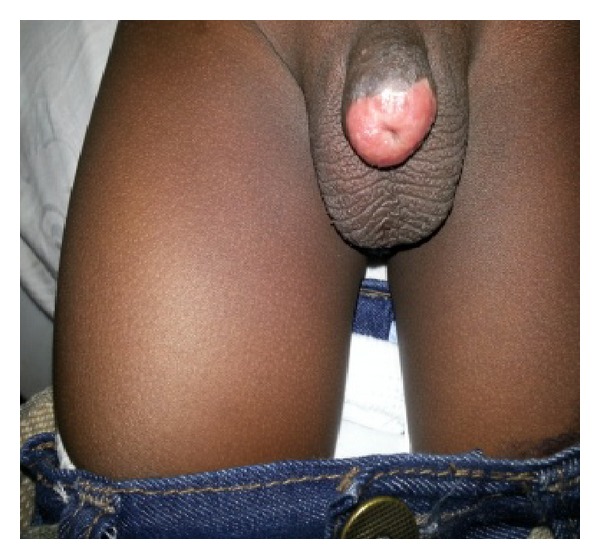
Glans penis at six months (AP view).

The graft improved in cosmetic appearance over time (Figures 6 and 7). The donor site healed within two weeks with no morbidity. At six-month follow-up, patient had a widely patent urethral meatus with no scarring at the distal end of the penis with acceptable cosmesis (Figures 8 and 9).

## 5. Discussion

Penile glans amputation like many others is a preventable complication of circumcision if proper attention is paid to detail and if it is carried out by properly trained personnel [2, 12, 13].

Among other factors, the management of penile glans amputation depends on the duration before presentation and the acute phase management usually involves autotransplantation [3–10]. In the first eight hours following circumcision, autotransplantation of the properly preserved glanular tissue is possible with significantly high success rate [2, 4–6]. Unfortunately, most of the penile amputations seen in our environment are reported very late far beyond the eight hours making any hope of graft survival slim.

Pack and Ariel described a technique in which the penile skin is brought over the ends of the corpora bodies and sutured to the urethra after partial penectomy [14]. This technique does not attempt to reconstruct the glans penis; thus, cosmesis is not guaranteed. It has a 6% chance of meatal stenosis and risk of penile shortening.

Skin grafts have also been used to fashion out a neoglans penis after partial penile amputation or used for resurfacing the glans after tumor excision [15, 16]. However, in a black skin, reconstructing a neoglans with skin grafts may not achieve satisfactory cosmetic results as the neoglans may blend with the penile shaft skin. Belinky et al. described the use of urethral flaps to reconstruct neoglans penis with acceptable cosmesis in ten patients with partial penile amputations for penile cancers [17]. This technique may require a long urethra and may be associated with penile curvature and shortening.

Scrotal flaps have also been used for glanuloplasty after partial penectomy with satisfactory penile function and appearance [12]. Again this technique has a high chance of stricture formation at the anastomotic site and hair may grow in the urethra over time despite laborious depilation at surgery. It is also a two-stage procedure.

Pedicled myocutaneous flaps based on the inferior epigastric artery have also been used to reconstruct the glans penis after glans amputation [13, 18]. Unfortunately, this technique requires expertise in microsurgery and instruments and may not be applicable in resource poor centers. Flap necrosis and infection may also develop.

We opted for the Venkov and Slavov method [11] of glanular reconstruction because it is simple to perform; it is a one stage procedure, provides excellent cosmesis especially in blacks, and can easily be done in resource poor centers.

One potential problem with this method of glanuloplasty is the high risk of graft mobility at the recipient site which can hinder neovascularization for adequate graft adaptation. We prevented this by using anchoring stitches. Another potential problem is graft contraction with resultant meatal stenosis. However, we did not observe this complication in our patient with a minimum of six-month follow-up.

## 6. Conclusion

Glanuloplasty with lower lip oral mucosa graft following total glans penis amputation from circumcision is simple and reproducible with satisfactory cosmetic and functional results and may be extended to the management of both traumatic and nontraumatic distal penile amputations.
